# Immunofluorescence deposits in the mesangial area and glomerular capillary loops did not affect the prognosis of immunoglobulin a nephropathy except C1q:a single-center retrospective study

**DOI:** 10.1186/s12882-021-02237-w

**Published:** 2021-01-29

**Authors:** Lingzhi Wu, Di Liu, Ming Xia, Guochun Chen, Yu Liu, Xuejing Zhu, Hong Liu

**Affiliations:** 1 Department of Nephrology, The Second Xiangya Hospital, Central South University, 139 Renmin Road, Changsha, 410011 Hunan China; 2 Hunan Key Laboratory of Kidney Disease and Blood Purification, 139 Renmin Road, Changsha, 410011 Hunan China

**Keywords:** IgA nephropathy, Immunofluorescence study, Prognosis

## Abstract

**Background:**

Immunoglobulin A nephropathy (IgAN) is identified as mesangial IgA deposition and is usually accompanied by other immunofluorescence deposits. The impact of immunofluorescent features in IgAN patients, however, remains unclear.

**Methods:**

Baseline clinicopathologic parameters and renal outcomes of 337 patients diagnosed with IgAN between January 2009 and December 2015 were analyzed. We then categorized these patients into four groups: without immunofluorescence deposits, mesangial-only, mesangial and glomerular capillary loops (GCLs), and GCLs-only. The study endpoint was end-stage kidney disease (ESKD) or a ≥ 50% decline in the estimated glomerular filtration rate (eGFR). Kaplan–Meier and Cox regression analyses were performed to calculate renal survival.

**Results:**

Of the 337 IgAN patients, women comprised 57.0%. Compared to patients with IgA deposition in the mesangial-only group, patients with IgA deposition in the mesangial +GCLs group were much heavier, and exhibited higher systolic blood pressure, lower serum IgG levels, and heavier proteinuria (all *P* < 0.05). Patients with IgG deposition in the mesangial +GCLs group presented with higher levels of cholesterol, heavier proteinuria than IgG deposition in the mesangial-only group (both *P* < 0.05). Compared with the mesangial-only group exhibiting C3 deposits, patients in the mesangial +GCLs group with C3 deposition had a higher systolic blood pressure (*P* = 0.028). A total of 38 patients (11.3%) continued to the study endpoint after a median follow-up time of 63.5 months (range,49.8–81.4). Kaplan–Meier analysis and Cox regression analysis showed that C1q deposition in the mesangial +GCLs group predicted a poor renal prognosis.

**Conclusions:**

IgA and IgG deposits in the mesangial region and GCLs were associated with more unfavorable clinical and histopathologic findings in IgAN patients. C1q deposition in the mesangial region and GCLs predicted a poor renal prognosis. However, the impact of the pattern of immunofluorescence deposits on renal outcomes remains to be proven by further investigation.

## Background

Immunoglobulin A (IgA) nephropathy (IgAN)—the most prevalent primary glomerular disease worldwide—can progress to kidney failure. Over the past 20–30 years, 10–30% of patients have developed end-stage kidney disease (ESKD) [1, 2]. Therefore, it is of paramount importance to obtain personalized risk predictions. Previous observations have suggested some specific risk predictors for the prognosis of IgAN [2]. Of these observations, proteinuria, abnormal blood pressure, and renal insufficiency at baseline are now widely recognized [3, 4]. Although the newest version of the Oxford classification system for IgAN has been used increasingly in clinical settings [5], immunofluorescent features were unfortunately excluded.

We have previously detected IgG, IgM, C3, and fibrinogen (Fib) (and rarely C1q) deposits in the mesangial region and/or glomerular capillary loops (GCLs) in addition to mesangial IgA deposition. A higher intensity of immunofluorescence deposits was associated with worsened clinical characteristics and poor prognoses of IgAN [6]. However, the relationships between the site of immunofluorescence and the clinicopathologic features and prognoses of IgAN remain unelucidated.

Therefore, we conducted a retrospective study to investigate the roles played by the types and patterns of immunofluorescence deposits in IgAN.

## Methods

### Subjects and study design

For this retrospective single-center study, we enrolled patients who were diagnosed with IgAN by kidney biopsy between January 2009 and December 2015 at the Second Xiangya Hospital of Central South University. Our exclusion criteria were (1) secondary IgAN alone or combined with other diseases—such as IgA vasculitis with nephritis, cirrhosis, lupus nephritis, diabetes, or tumors; (2) patients with acute infection or concerns regarding a diagnosis with infection-associated glomerulonephritis; (3) an estimated glomerular filtration rate (eGFR) below 15 ml/min/1.73 m^2^ at admission; and (4) glomerular number < 8 and cortical thickness < 5 mm. Our study ultimately included 337 patients, and after the median follow-up time of 63.5 months (49.8, 81.4), 38 (11.3%) patients reached the final renal endpoint.

### Data collection

We recorded clinical and experimental information of IgAN patients at admission and follow-up data, including general data (clinical history, sex, age, weight, blood pressure), blood biochemical indicators, and proteinuria.

### Kidney biopsy

Each kidney biopsy was routinely observed via light microscopy immunofluorescence, and electron microscopy by at least two pathologists [7]. For direct immunofluorescence, frozen specimens were reacted with polyclonal rabbit anti-human IgA, IgG, IgM, C1q, C3, or Fib and then examined under a fluorescence photomicroscope. We evaluated the intensity of immunofluorescence deposits semi-quantitatively. The patients were categorized based upon the type and pattern of immunofluorescence deposits (without IgG [or IgM, C3, Fib, or C1q] deposits, mesangial-only, mesangial+GCLs, or GCLs-only). The specimens from patients enrolled in our study were then reassessed according to the new version of the Oxford classification (MEST-C score system) by the same two pathologists [5]. Differences in scoring were resolved by a senior pathologist.

### Renal outcomes

The definition for the study endpoint was an ESKD or a ≥ 50% decline in the eGFR.

### Definitions

We defined a systolic blood pressure (SBP) ≥140 mmHg and/or diastolic blood pressure (DBP) ≥90 mmHg, or the use of drugs that control blood pressure, as hypertension. Mean arterial pressure (MAP) was calculated using DBP plus 1/3 of the pulse pressure. A level of hemoglobin below 110 g/L and 120 g/L (females and males, respectively) was defined as anemia. We evaluated the eGFR with the Chronic Kidney Disease Epidemiology Collaboration equation [8]. ESKD referred to an eGFR less of than 15 ml/min/1.73 m^2^ or treatment with renal-replacement therapy.

### Statistical analyses

We used SPSS 22.0 software. Means ± SD were used to express the data that conformed to a normal distribution, and t-test and 1-way ANOVA were used to test the comparison between/among groups. Data that were not normally distributed were expressed as a median (M, with lower quartiles, upper quartiles), and comparisons between groups were analyzed using the non-parametric rank-sum test, with post-hoc comparisons made using the Bonferroni method to correct significance levels. To calculate the cumulative renal survival, Kaplan–Meier analysis and Cox regression analysis were performed. Statistical differences were set at *P* < 0.05.

## Results

### Baseline data

Of the 337 patients investigated in our study, 57.0% were female, with a median age of 30.0 (range, 24.0–40.0) years. The prevalence of hypertension and anemia was found to be 24.6 and 16.0%, respectively. Other characteristics and the types and patterns of immunofluorescence deposits are shown in Tables 1 and 2.

**Table 1:** Baseline characteristics of 337 IgAN patients at diagnosis

Parameters	*N* = 337
Age (years)	30.0 (24.0, 40.0)
Female (n, %)	192 (57.0)
Weight (kg)	59.5 ± 11.2
Systolic blood pressure (mmHg)	125.8 ± 16.4
Diastolic blood pressure (mmHg)	80.4 ± 12.3
Mean arterial pressure (mmHg)	95.5 ± 12.7
Hypertension (%)	24.6
Anemia (%)	16.0
Hemoglobin (g/L)	130.3 ± 18.4
Serum albumin (g/L)	37.6 (33.3, 41.0)
eGFR (ml/min/1.73m^2^)	92.4 ± 32.3
Serum creatinine (μmol/L)	78.8 (60.2, 99.5)
Triglyceride (mmol/L)	1.9 ± 2.1
Cholesterol (mmol/L)	5.2 ± 2.2
Serum IgA (g/L)	2.7 ± 1.1
Serum IgG (g/L)	10.3 ± 3.3
Serum IgM (g/L)	1.4 ± 0.7
Serum C3 (g/L)	1.0 ± 0.3
Serum C4 (g/L)	0.3 ± 0.2
Proteinuria (g/24 h)	0.6 (0.3, 1.6)
Immunosuppression (%)	39.0
Corticosteroid (%)	20.3
Renin-angiotensin system blockade (%)	62.6
Tonsillectomy (%)	19.8
IgA Mesangial-only (n)	251
Mesangial +GCLs (n)	86
IgG (−) (n)	258
Mesangial-only (n)	32
Mesangial +GCLs (n)	33
GCLs-only (n)	14
IgM (−) (n)	199
Mesangial-only (n)	102
Mesangial +GCLs (n)	35
GCLs-only (n)	1
C3 (−) (n)	162
Mesangial-only (n)	127
Mesangial +GCLs (n)	46
GCLs-only (n)	2
Fib (−) (n)	299
Mesangial-only (n)	17
Mesangial +GCLs (n)	19
GCLs-only (n)	2
C1q (−) (n)	303
Mesangial-only (n)	19
Mesangial +GCLs (n)	14
GCLs-only (n)	1
M1 (%)	5.6
E1 (%)	7.4
S1 (%)	71.8
T (1 + 2) (%)	73.9
C (1 + 2) (%)	37.7

Abbreviations: *eGFR* estimated glomerular filtration rate, *C3* complement 3, *C4* complement 4, *IgG (−)* without IgG deposits, *IgM (−)* without IgM deposits, *C3(−)* without C3 deposits, *Fib (−)* without Fib deposits, *C1q (−)* without C1q deposits, *GCLs* glomerular capillary loops, *Fib* Fibrinogen, *M* mesangial hypercellularity, *E* endocapillary hypercellularity, *S* segmental glomerulosclerosis, *T* tubular atrophy/interstitial fibrosis, *C* Cellular or fibrocellular crescents

**Table 2:** The pattern of IgA staining co-existent with IgG, IgM, C3, Fib, C1q staining

Parameter	IgA	
	Mesangial-only (n)	Mesangial +GCLs (n)
IgG (+)	46	33
IgM (+)	100	38
C3 (+)	136	39
Fib (+)	23	15
C1q (+)	19	15

Abbreviations: *Fib* Fibrinogen, *IgG (+)* with IgG deposits, *IgM (+)* with IgM deposits, *C3 (+)* with C3 deposits, *Fib (+)* with Fib deposits, *C1q (+)* with C1q deposits, *GCLs* glomerular capillary loops

### Characteristics of patients with immunofluorescence deposits in the mesangial-only group

IgG deposition in the mesangial-only group exhibited a lower frequency of T(1 + 2) compared with patients without IgG deposition (*P* = 0.044). Compared with the patients without IgM staining, those patients showing IgM deposition in the mesangial-only group showed a higher serum concentration of IgM and a lower percentage of crescents (*P* < 0.001, *P* = 0.034). The patients with mesangial staining for C3 presented with lower serum C3 levels relative to those without C3 deposition (*P* = 0.005). The mesangial staining for C1q was significantly associated with heavier proteinuria compared with C1q-negative staining (*P* = 0.010). There were no significant differences in the baseline characteristics between patients without Fib deposition and those with Fib deposition in the mesangial-only group (Tables 3, 4, 5 and 6).

**Table 3:** Comparison of general data according to the location of immunofluorescence deposits at diagnosis

Parameters	N	Age (years)	Female (n, %)	Weight (kg)	SBP (mmHg)	DBP (mmHg)	MAP (mmHg)
IgA Mesangial-only	251	30.0 (24.0,39.0)	143 (56.9)	58.7 ± 10.8	124.6 ± 16.2	79.9 ± 12.5	94.8 ± 12.7
Mesangial +GCLs	86	32.0 (23.0,42.2)	49 (57.0)	61.6 ± 12.1^*^	129.2 ± 16.8^*^	81.8 ± 11.8	97.6 ± 12.4
*P*		0.697	0.999	0.040	0.037	0.223	0.077
IgG (−)	258	30.0 (24.0,39.0)	146 (56.5)	59.4 ± 11.2	125.5 ± 16.6	80.1 ± 13.0	95.2 ± 13.1
Mesangial-only	32	29.0 (24.2,40.7)	17 (53.1)	59.0 ± 10.6	123.9 ± 15.4	79.7 ± 10.1	94.4 ± 11.0
Mesangial +GCLs	33	32.0 (21.0,45.5)	22 (66.7)	60.9 ± 13.3	130.0 ± 18.2	83.2 ± 10.5	98.8 ± 12.1
*P*		0.962	0.482	0.897	0.329	0.277	0.293
IgM (−)	199	31.0 (24.0,40.0)	106 (53.3)	59.1 ± 11.0	125.5 ± 16.3	80.2 ± 12.6	95.3 ± 12.8
Mesangial-only	102	29.5 (24.0,39.2)	63 (61.8)	60.0 ± 11.7	125.2 ± 17.0	79.9 ± 11.6	95.0 ± 12.3
Mesangial +GCLs	35	29.0 (24.0,39.0)	22 (62.8)	60.0 ± 11.3	129.2 ± 16.0	82.1 ± 12.6	97.8 ± 13.1
*P*		0.981	0.279	0.729	0.407	0.736	0.554
C3 (−)	162	31.5 (23.0,40.2)	86 (53.1)	60.3 ± 11.8	126.0 ± 16.7	80.7 ± 12.5	95.8 ± 12.9
Mesangial-only	127	29.0 (24.0,37.0)	78 (61.4)	58.5 ± 10.5	123.6 ± 15.5	78.6 ± 11.4	93.6 ± 11.5
Mesangial +GCLs	46	36.5 (24.0,40.5)	26 (56.5)	59.5 ± 11.3	131.2 ± 17.4^**b**^	83.9 ± 13.8	99.7 ± 14.3
*P*		0.619	0.367	0.390	0.028	0.110	0.052
Fib (−)	299	29.0 (24.0,38.0)	171 (57.2)	59.2 ± 11.0	125.6 ± 16.0	80.2 ± 12.5	95.3 ± 12.6
Mesangial-only	17	39.0 (30.0,41.0)	11 (64.7)	59.0 ± 11.0	124.0 ± 18.7	83.1 ± 10.6	96.7 ± 12.7
Mesangial +GCLs	19	39.0 (23.0,44.0)	9 (47.4)	63.4 ± 13.9	131.8 ± 20.8	82.0 ± 11.6	98.6 ± 14.1
*P*		0.068	0.568	0.469	0.318	0.345	0.512
C1q (−)	303	30.0 (24.0,39.0)	175 (57.7)	59.2 ± 11.0	125.1 ± 15.7	80.1 ± 12.0	95.1 ± 12.2
Mesangial-only	19	35.0 (24.0,42.0)	10 (52.6)	61.2 ± 12.2	130.8 ± 24.3	80.8 ± 14.0	97.5 ± 16.6
Mesangial +GCLs	14	27.5 (24.5,43.2)	7 (50.0)	62.7 ± 14.0	133.2 ± 17.8	86.1 ± 16.0	101.8 ± 16.3
*P*		0.865	0.781	0.392	0.079	0.368	0.214

Abbreviations: *SBP* systolic blood pressure, *DBP* diastolic blood pressure, *MAP* mean arterial pressure, *Fib* Fibrinogen, *IgG (−)* without IgG deposits, *IgM (−)* without IgM deposits, *C3(−)* without C3 deposits, *Fib (−)* without Fib deposits, *C1q (−)* without C1q deposits, *GCLs* glomerular capillary loops

* *P* compare between the IgA groups

^a^ *P* compare with (−) group

^b^ *P* compare with the Mesangial-only group

Statistically significant at *P* < 0.05

The patients with immune deposits in the GCLs-only group were excluded due to the small sample size

**Table 4:** Comparison of laboratory data according to the location of immunofluorescence deposits at diagnosis

Parameter		N	Hemoglobin (g/L)	Serum albumin (g/L)	eGFR (ml/min/1.73m^2^)	Scr (μmol/L)	TG (mmol/L)	CHOL (mmol/L)	Serum IgA (g/L)	Serum IgG (g/L)	Serum IgM (g/L)	Serum C3 (g/L)	Proteinuria (g/24 h)
IgA	Mesangial-only	251	130.1 ± 18.1	37.7 (33.7,41.1)	92.4 ± 31.9	78.2 (59.2,78.2)	1.9 ± 2.1	5.1 ± 2.2	2.7 ± 1.1	10.4 ± 3.3	1.4 ± 0.7	1.0 ± 0.3	0.5 (0.3,1.5)
	Mesangial +GCLs	86	130.9 ± 19.2	36.7 (31.3,40.5)	92.3 ± 33.3	80.2 (62.1,103.0)	2.0 ± 1.9	5.5 ± 2.2	2.8 ± 1.1	9.8 ± 3.2^*^	1.3 ± 0.6	1.0 ± 0.2	1.1 (0.5,1.9) ^*^
	*P*		0.925	0.276	0.754	0.454	0.607	0.077	0.896	0.048	0.266	0.125	< 0.001
IgG	(−)	258	130.3 ± 18.6	37.6 (33.7,41.1)	91.3 ± 31.2	79.3 (60.3,102.8)	2.0 ± 2.3	5.3 ± 2.3	2.7 ± 1.1	10.2 ± 3.2	1.4 ± 0.7	1.0 ± 0.3	0.6 (0.3,1.6)
	Mesangial-only	32	132.4 ± 18.7	37.9 (34.1,40.6)	101.6 ± 34.5	73.0 (58.4,92.3)	1.4 ± 0.8	4.6 ± 2.0	2.9 ± 1.2	11.0 ± 3.2	1.5 ± 0.8	0.9 ± 0.3	0.4 (0.2,0.7)
	Mesangial +GCLs	33	128.5 ± 17.2	35.8 (28.8,41.3)	94.9 ± 37.6	70.6 (59.5,95.6)	1.7 ± 1.3	5.6 ± 2.0^**b**^	2.8 ± 1.2	9.9 ± 3.6	1.4 ± 0.7	1.0 ± 0.2	1.2 (0.4,3.1) ^**b**^
	*P*		0.646	0.758	0.382	0.289	0.325	0.044	0.488	0.406	0.742	0.157	0.008
IgM	(−)	199	129.0 ± 18.4	37.6 (33.7,41.1)	89.7 ± 32.0	80.5 (62.6,102.8)	1.7 ± 1.5	5.1 ± 2.1	2.6 ± 1.0	10.2 ± 3.2	1.2 ± 0.6	1.0 ± 0.2	0.6 (0.3,1.6)
	Mesangial-only	102	132.8 ± 17.6	36.4 (32.6,41.2)	97.9 ± 32.2	72.2 (58.3,95.4)	2.4 ± 3.0	5.4 ± 2.5	2.8 ± 1.1	10.2 ± 3.3	1.6 ± 0.8^**a**^	1.1 ± 0.3	0.6 (0.3,1.5)
	Mesangial +GCLs	35	130.8 ± 20.2	37.1 (33.7,40.6)	92.5 ± 32.3	74.3 (59.9,103.3)	1.8 ± 1.4	5.2 ± 2.1	3.0 ± 1.3	11.1 ± 3.8	1.5 ± 0.7	1.0 ± 0.3	0.6 (0.4,1.9)
	*P*		0.332	0.946	0.075	0.144	0.347	0.931	0.174	0.308	< 0.001	0.246	0.565
C3	(−)	162	131.5 ± 18.7	37.9 (31.2,41.2)	91.9 ± 31.5	79.4 (62.6,99.0)	1.8 ± 1.5	5.4 ± 2.4	2.6 ± 1.1	10.1 ± 3.5	1.4 ± 0.8	1.0 ± 0.2	0.6 (0.3,1.6)
	Mesangial-only	127	129.1 ± 17.2	37.7 (33.9,41.1)	94.4 ± 34.1	77.6 (56.4,99.1)	2.1 ± 2.7	5.1 ± 2.3	2.8 ± 1.1	10.5 ± 2.9	1.4 ± 0.7	1.0 ± 0.3^**a**^	0.5 (0.3,1.3)
	Mesangial +GCLs	46	129.7 ± 20.7	35.5 (33.1,39.5)	89.5 ± 30.5	79.8 (60.3,104.4)	1.8 ± 1.6	5.0 ± 1.4	2.8 ± 1.0	10.5 ± 3.3	1.3 ± 0.7	1.0 ± 0.2	1.0 (0.4,1.5)
	*P*		0.291	0.321	0.673	0.541	0.662	0.297	0.147	0.723	0.466	0.005	0.102
Fib	(−)	299	129.7 ± 18.3	37.6 (32.6,41.1)	92.3 ± 33.5	79.3 (59.4,100.8)	1.9 ± 2.0	5.3 ± 2.3	2.7 ± 1.1	10.2 ± 3.3	1.4 ± 0.7	1.0 ± 0.3	0.6 (0.3,1.6)
	Mesangial-only	17	132.2 ± 17.9	37.0 (33.8,38.7)	87.6 ± 17.3	73.9 (63.4,89.2)	1.7 ± 1.0	4.4 ± 1.2	3.2 ± 0.9	11.4 ± 3.4	1.3 ± 0.7	0.9 ± 0.2	0.5 (0.3,1.4)
	Mesangial +GCLs	19	139.5 ± 17.4	37.9 (34.4,41.1)	95.2 ± 23.7	70.6 (60.4,99.9)	2.9 ± 3.1	5.2 ± 1.3	2.9 ± 1.4	10.0 ± 2.7	1.1 ± 0.4	1.1 ± 0.2	0.7 (0.5,1.7)
	*P*		0.168	0.772	0.747	0.819	0.363	0.388	0.126	0.337	0.453	0.079	0.461
C1q	(−)	303	130.0 ± 18.5	37.6 (33.6,41.1)	93.2 ± 31.5	78.0 (59.3,98.2)	1.8 ± 1.8	5.2 ± 2.2	2.7 ± 1.0	10.3 ± 3.2	1.4 ± 0.7	1.0 ± 0.3	0.6 (0.3,1.5)
	Mesangial-only	19	134.7 ± 16.4	35.6 (31.2,39.2)	81.9 ± 31.4	85.4 (66.1,138.7)	3.3 ± 4.8	5.4 ± 1.8	2.8 ± 1.6	9.3 ± 3.7	1.5 ± 0.9	1.1 ± 0.5	1.2 (0.6,3.8) ^**a**^
	Mesangial +GCLs	14	131.3 ± 19.2	38.3 (32.5,41.1)	92.2 ± 46.1	91.1 (60.1,146.3)	2.0 ± 1.4	5.3 ± 2.4	3.0 ± 1.3	11.2 ± 4.0	1.3 ± 0.8	1.1 ± 0.2	1.1 (0.4,2.6)
	*P*		0.586	0.400	0.269	0.185	0.159	0.755	0.820	0.317	0.708	0.391	0.010

Abbreviations: *eGFR* estimated glomerular filtration rate, *Scr* serum creatinine, *TG* triglyceride, *CHOL* cholesterol, *C3* complement 3, *Fib* Fibrinogen, *IgG (−)* without IgG deposits, *IgM (−)* without IgM deposits, *C3(−)* without C3 deposits, *Fib (−)* without Fib deposits, *C1q (−)* without C1q deposits, *GCLs* glomerular capillary loops

* *P* compare between the IgA groups

^a^ *P* compare with (−) group

^b^ *P* compare with the Mesangial-only group

Statistically significant at *P* < 0.05

The patients with immune deposits in the GCLs group were excluded due to the small sample size

**Table 5:** Comparison of light microscopic findings according to the location of immunofluorescence deposits at diagnosis

Parameters		N	Segmental glomerulosclerosis (%)	Global glomerulosclerosis (%)	Crescent (cellular or fibrocellular) (%)
IgA	Mesangial-only	251	9.1 (0.0,18.2)	12.5 (3.2,27.3)	0.0 (0.0,6.4)
	Mesangial +GCLs	86	11.9 (4.0,20.3)	13.3 (5.5,30.8)	0.0 (0.0,9.5)
	*P*		0.094	0.469	0.107
IgG	(−)	258	10.0 (0.0,18.8)	14.3 (0.0,30.0)	0.0 (0.0,7.7)
	Mesangial-only	32	7.5 (0.0,14.1)	9.7 (0.0,29.3)	0.0 (0.0,6.1)
	Mesangial +GCLs	33	9.1 (3.9,17.9)	9.5 (4.2,20.9)	0.0 (0.0,9.8)
	*P*		0.569	0.350	0.665
IgM	(−)	199	10.0 (0.0,18.7)	12.5 (4.0,27.6)	0.0 (0.0,8.3)
	Mesangial-only	102	7.7 (0.0,16.2)	12.5 (0.0,27.6)	0.0 (0.0,4.0) ^**a**^
	Mesangial +GCLs	35	15.4 (6.7,21.4)	18.9 (6.2,29.4)	0.0 (0.0,7.7)
	*P*		0.113	0.534	0.034
C3	(−)	162	9.3 (0.0,19.6)	12.5 (4.7,27.2)	0.0 (0.0,7.3)
	Mesangial-only	127	8.3 (0.0,15.4)	12.5 (0.0,29.0)	0.0 (0.0,6.7)
	Mesangial +GCLs	46	12.5 (6.0,20.0)	15.6 (6.6,31.4)	0.0 (0.0,9.6)
	*P*		0.193	0.525	0.685
Fib	(−)	299	10.0 (0.0,18.2)	12.5 (4.0,28.6)	0.0 (0.0,7.7)
	Mesangial-only	17	10.0 (5.6,26.7)	15.0 (4.0,27.5)	0.0 (0.0,7.0)
	Mesangial +GCLs	19	9.1 (0.0,17.6)	20.0 (8.0,24.1)	0.0 (0.0,5.3)
	*P*		0.526	0.833	0.836
C1q	(−)	303	9.5 (0.0,18.2)	12.5 (3.7,27.3)	0.0 (0.0,7.7)
	Mesangial-only	19	15.4 (4.8,25.0)	10.0 (6.2,33.3)	0.0 (0.0,4.0)
	Mesangial +GCLs	14	8.2 (0.0,19.5)	17.7 (11.8,50.2)	0.0 (0.0,1.9)
	*P*		0.226	0.244	0.520

Abbreviations: *Fib* Fibrinogen, *IgG (−)* without IgG deposits, *IgM (−)* without IgM deposits, *C3(−)* without C3 deposits, *Fib (−)* without Fib deposits, *C1q (−)* without C1q deposits, *GCLs* glomerular capillary loops

* *P* compare between the IgA groups

^a^ *P* compare with (−) group

^b^ *P* compare with the Mesangial-only group

Statistically significant at *P* < 0.05

The patients with immune deposits in the GCLs group were excluded due to the small sample size

**Table 6:** Comparison of the Oxford classification according to the location of immunofluorescence deposits at diagnosis

Parameters		N	M1 (%)	E1 (%)	S1 (%)	T (1 + 2) (%)	C (1 + 2) (%)
IgA	Mesangial-only	251	4.8	7.6	69.7	73.3	35.9
	Mesangial +GCLs	86	5.6	7.0	77.9	73.9	43.0
	*P*		0.244	0.856	0.145	0.679	0.237
IgG	(−)	258	4.7	7.8	71.7	76.4	36.0
	Mesangial-only	32	6.3	9.4	68.8	56.3 ^**a**^	43.8
	Mesangial +GCLs	33	15.2 ^**a**^	6.1	78.8	69.7	42.4
	*P*		0.037	0.882	0.627	0.044	0.575
IgM	(−)	199	6.0	9.5	69.8	77.9	42.2
	Mesangial-only	102	3.9	5.9	70.6	66.7	28.4
	Mesangial +GCLs	35	8.6	0.0	85.7	71.4	37.1
	*P*		0.553	0.108	0.150	0.105	0.065
C3	(−)	162	4.9	8.6	69.8	74.7	34.6
	Mesangial-only	127	4.7	6.3	70.9	74.8	40.2
	Mesangial +GCLs	46	10.9	6.5	80.4	69.6	41.3
	*P*		0.259	0.728	0.355	0.758	0.533
Fib	(−)	299	6.4	8.0	70.9	75.3	36.8
	Mesangial-only	17	0.0	0.0	82.4	64.7	47.1
	Mesangial +GCLs	19	0.0	5.3	73.7	57.9	36.8
	*P*		0.297	0.440	0.583	0.171	0.695
C1q	(−)	303	5.3	7.6	71.0	74.3	38.3
	Mesangial-only	19	5.3	10.5	84.2	68.4	36.8
	Mesangial +GCLs	14	7.1	0.0	71.4	71.4	21.4
	*P*		0.955	0.497	0.461	0.836	0.444

Abbreviations: *Fib* Fibrinogen, *IgG (−)* without IgG deposits, *IgM (−)* without IgM deposits, *C3(−)* without C3 deposits, *Fib (−)* without Fib deposits, *C1q (−)* without C1q deposits, *GCLs* glomerular capillary loops, *M* mesangial hypercellularity, *E* endocapillary hypercellularity, *S* segmental glomerulosclerosis, *T* tubular atrophy/interstitial fibrosis, *C* Cellular or fibrocellular crescents

* *P* compare between the IgA groups

^a^ *P* compare with (−) group

^b^ *P* compare with the Mesangial-only group

Statistically significant at *P* < 0.05

The patients with immune deposits in the GCLs group were excluded due to the small sample size

### Characteristics of patients with immunofluorescence deposits in the mesangial+GCLs group

Compared with patients with IgA deposition in the mesangial-only group, patients with IgA deposition in the mesangial+GCLs group were much heavier, had higher SBP, lower serum IgG levels, and heavier proteinuria (all *P* < 0.05). Patients with IgG deposition in the mesangial +GCLs group presented with higher levels of cholesterol (CHOL), heavier proteinuria than IgG deposition in the mesangial-only group (*P* = 0.044, *P* = 0.008, respectively). Patients with IgM deposition in the mesangial+GCLs group showed a tendency toward higher serum IgM levels; however, no difference was uncovered between the mesangial+GCLs group and the mesangial-only group with respect to IgM. Compared to patients with C3 deposits in the mesangial-only group, patients with C3 deposition in the mesangial+GCLs group manifested a higher SBP (*P* = 0.028). We found no statistical differences in baseline characteristics between the patients with or without Fib deposition in the GCLs, as was C1q (Tables 3, 4, 5, and 6).

### The relationship between the intensity and pattern of immunofluorescence deposits

The relationship between the intensity and pattern of immunofluorescence deposits is shown in Table 7. Patients with IgA deposits in the mesangial+GCLs group presented more commonly with IgG, Fib, and C1q co-deposition than those with IgA deposits restricted to the mesangial area. Interestingly, the pattern of IgG deposition related closely to the intensity of IgA deposits. Patients with IgG deposition in the mesangial-only group usually had an IgA deposition intensity ≥3+, while those with IgG deposition in the mesangial+GCLs group exhibited a more even distribution of IgA-deposition intensity (*P* = 0.024). Approximately half of the patients with IgG deposition in the mesangial-only group demonstrated a C3 deposition intensity ≥2+, while over half of the patients with IgG deposition in the mesangial +GCLs group had no C3 deposition. IgG deposition in the mesangial+GCLs group was more likely to be found co-deposited with Fib and C1q. IgM deposition in the mesangial-only group was also closely related to the higher intensity of IgA deposition (*P* = 0.007). IgM deposition in the mesangial+GCLs group was also more likely to co-deposit with Fib and C1q. IgA-deposition intensity was marked in both the mesangial+GCLs and mesangial-only groups with C3 deposits (*P* < 0.001). We observed that patients with C3 deposits in the mesangial+GCLs group more frequently presented with IgG, IgM, Fib, and C1q co-deposition (all *P* < 0.05). The pattern of Fib deposits was related to the intensity of IgA deposits and the presence of IgG, IgM, and C1q deposits (all *P* < 0.05). The location of C1q deposits was also associated with the intensity of IgA staining and the presence of IgG, IgM, and Fib deposits (all *P* < 0.05).

**Table 7:** The association of immunofluorescence deposits

Parameter		N	IgA (n, %)			IgG (n, %)	IgM (n, %)	C3 (n, %)			Fib (n, %)	C1q (n, %)
			1+	2+	≥3+	(+)	(+)	(−)	<2+	≥ 2+	(+)	(+)
IgA	Mesangial-only	251	51 (20.3)	78 (31.1)	122 (48.6)	46 (18.3)	100 (39.8)	115 (45.8)	42 (16.7)	94 (37.5)	23 (9.2)	19 (7.6)
	Mesangial +GCLs	86	18 (20.9)	25 (29.1)	43 (50.0)	33 (38.4) ^*^	38 (44.2)	47 (54.7)	12 (14.0)	27 (31.4)	15 (17.4) ^*^	15 (17.4) ^*^
	*P*		0.941			< 0.001	0.479	0.367			0.036	0.009
IgG	(−)	258	54 (20.9)	83 (32.2)	121 (46.9)		104 (40.3)	122 (47.3)	43 (16.7)	93 (36.0)	23 (8.9)	20 (7.8)
	Mesangial-only	32	1 (3.1) ^**a**^	9 (28.1)	22 (68.8)		17 (53.1)	12 (37.5)	3 (9.4)	17 (53.1)	6 (18.8)	4 (12.5)
	Mesangial +GCLs	33	7 (21.2)	7 (21.2)	19 (57.6)		14 (42.4)	17 (51.5)	6 (18.2)	10 (30.3)	8 (24.2) ^**a**^	9 (27.3) ^**a**^
	*P*		0.024				0.383	0.232			0.013	0.002
IgM	(−)	199	50 (25.1)	64 (32.2)	85 (42.7)	45 (22.6)		105 (52.8)	33 (16.6)	61 (30.7)	12 (6.0)	11 (5.5)
	Mesangial-only	102	15 (14.7) ^**a**^	27 (26.5)	60 (58.8)	20 (19.6)		44 (43.1)	14 (13.7)	44 (43.1)	17 (16.7) ^**a**^	12 (11.8)
	Mesangial +GCLs	35	3 (8.6)	12 (34.3)	20 (57.1)	13 (37.1)		13 (37.1)	6 (17.1)	16 (45.7)	9 (25.7) ^**a**^	11 (31.4) ^**ab**^
	*P*		0.007			0.101		0.053			< 0.001	< 0.001
C3	(−)	162	58 (35.8)	55 (34.0)	49 (30.2)	40 (24.7)	57 (35.2)				15 (9.3)	15 (9.3)
	Mesangial-only	127	10 (7.9) ^**a**^	35 (27.6)	82 (64.6)	18 (14.2)	54 (42.5)				9 (7.1)	6 (4.7)
	Mesangial +GCLs	46	0 (0.0) ^**a**^	13 (28.3)	33 (71.7)	19 (41.3) ^**b**^	26 (56.5) ^**a**^				14 (30.4) ^**ab**^	13 (28.3) ^**ab**^
	*P*		< 0.001			0.001	0.031				< 0.001	< 0.001
Fib	(−)	299	67 (22.4)	96 (32.1)	136 (45.5)	64 (21.4)	112 (37.5)	147 (49.2)	48 (16.1)	104 (34.8)		24 (8.0)
	Mesangial-only	17	2 (11.8)	3 (17.6)	12 (70.6)	5 (29.4)	11 (64.7)	10 (58.8)	2 (11.8)	5 (29.4)		2 (11.8)
	Mesangial +GCLs	19	0 (0.0) ^**a**^	4 (21.1)	15 (78.9)	9 (47.4) ^**a**^	14 (73.7) ^**a**^	5 (26.3)	4 (21.1)	10 (52.6)		8 (42.1) ^**ab**^
	*P*		0.002			0.029	0.001	0.117				< 0.001
C1q	(−)	303	66 (21.8)	94 (31.0)	143 (47.2)	65 (21.5)	115 (38.0)	147 (48.5)	47 (15.5)	109 (36.0)	28 (9.2)	
	Mesangial-only	19	3 (15.8)	6 (31.6)	10 (52.6)	5 (26.3)	12 (63.2)	11 (57.9)	2 (10.5)	6 (31.6)	5 (26.3)	
	Mesangial +GCLs	14	0 (0.0) ^**a**^	3 (21.4)	11 (78.6)	8 (57.1) ^**a**^	11 (78.6) ^**a**^	4 (28.6)	5 (35.7)	5 (35.7)	5 (35.7) ^**a**^	
	*P*		0.041			0.008	0.001	0.530			0.001	

Abbreviations: *Fib* Fibrinogen, *IgG (−)* without IgG deposits, *IgM (−)* without IgM deposits, *C3(−)* without C3 deposits, *Fib (−)* without Fib deposits, *C1q (−)* without C1q deposits, *GCLs* glomerular capillary loops

* *P* compare between the IgA groups

^a^ *P* compare with (−) group

^b^ *P* compare with the Mesangial-only group

Statistically significant at *P* < 0.05

The patients with immune deposits in the GCLs group were excluded due to small sample size

### Correlation between renal outcomes and the pattern of immunofluorescence deposits

**Fig. 1:**
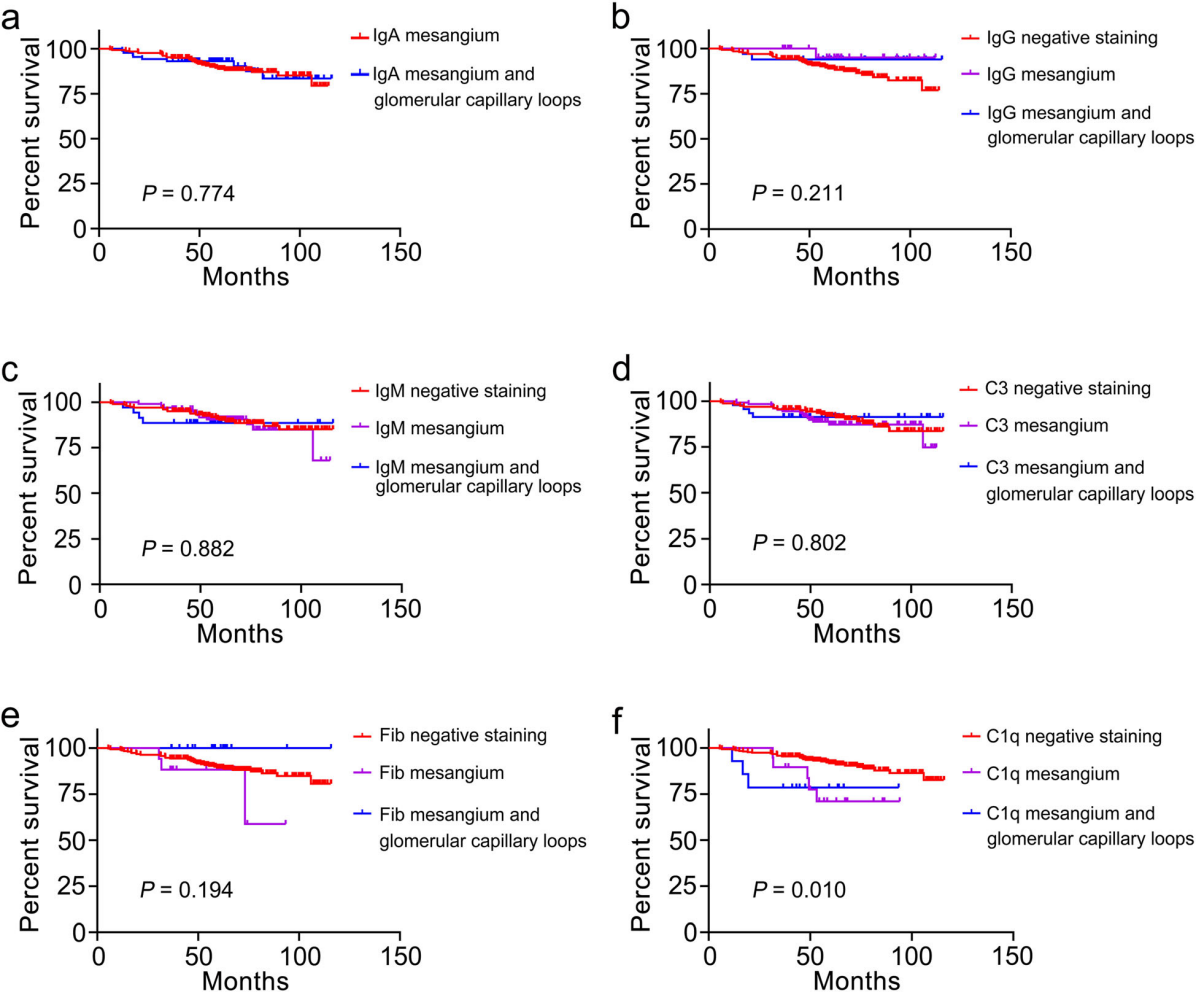
Cumulative renal survival and risk factors in patients with IgAN

After the median follow-up time of 63.5 (49.8, 81.4) months, 38 (11.3%) patients reached renal outcome. Our results showed that the follow-up time was shortest in patients with C1q deposition in the mesangial+GCLs group, and that there were also statistical differences in follow-up time among the C3 groups (shown in Table 8). Although the location of C1q deposition was associated with renal prognosis according to Kaplan–Meier analysis (*P* < 0.05), we determined no other significant association between any other immunostaining pattern and cumulative renal survival (all *P* > 0.05) (Fig. 1). Importantly, multivariate Cox regression analysis showed that C1q deposition in the mesangial+GCLs predicted a poor renal prognosis (Table 9).

**Table 8:** Comparison of the treatment at diagnosis and follow-up data according to the location of immunofluorescence deposits

Parameter		N	Corticosteroid (%)	Immunosuppression (%)	RAS blockade (%)	Follow-up time (months)	ESKD (n, %)
IgA	Mesangial-only	251	21.7	37.8	65.0	64.3 (49.9,81.2)	29 (11.6)
	Mesangial +GCLs	86	15.9	43.2	54.5	61.3 (48.4,83.1)	9 (10.5)
	*P*		0.406	0.519	0.209	0.827	0.783
IgG	(−)	258	20.6	39.7	65.2	62.5 (50.5,81.3)	33 (12.8)
	Mesangial-only	32	38.5	23.1	53.8	59.9 (39.5,68.8)	1 (3.1)
	Mesangial +GCLs	33	9.5	47.6	57.1	64.8 (51.9,93.8)	2 (6.1)
	*P*		0.128	0.357	0.585	0.213	0.069
IgM	(−)	199	18.6	39.8	62.7	65.7 (49.7,88.1)	21 (10.6)
	Mesangial-only	102	25.9	37.9	67.2	64.4 (54.4,78.8)	12 (11.8)
	Mesangial +GCLs	35	0.0	30.0	40.0	57.5 (46.6,66.5)	4 (11.4)
	*P*		0.143	0.820	0.257	0.108	0.762
C3	(−)	162	20.4	43.4	70.8	73.2 (56.3,87.1)	18 (11.1)
	Mesangial-only	127	20.3	30.5	50.8 ^**a**^	55.9 (48.1,74.7) ^**a**^	15 (11.8)
	Mesangial +GCLs	46	14.3	35.7	50.0	58.5 (41.0,69.0) ^**a**^	4 (8.7)
	*P*		0.861	0.252	0.021	< 0.001	0.829
Fib	(−)	299	21.8	37.9	61.5	65.0 (50.4,86.0)	35 (11.7)
	Mesangial-only	17	0.0	80.0	100.0	59.4 (36.6,66.8)	3 (17.6)
	Mesangial +GCLs	19	0.0	33.3	66.7	58.4 (47.2,64.3)	0 (0.0)
	*P*		0.221	0.157	0.210	0.032**#**	0.487
C1q	(−)	303	19.7	38.7	63.6	64.8 (51.1,86.0)	30 (9.9)
	Mesangial-only	19	40.0	50.0	60.0	56.8 (48.6,68.3)	5 (26.3)
	Mesangial +GCLs	14	0.0	25.0	25.0	46.2 (32.4,64.7) ^**a**^	3 (21.4)
	*P*		0.177	0.656	0.284	0.011	0.043**#**

Abbreviations: *Fib* Fibrinogen, *IgG (−)* without IgG deposits, *IgM (−)* without IgM deposits, *C3(−)* without C3 deposits, *Fib (−)* without Fib deposits, *C1q (−)* without C1q deposits, *GCLs* glomerular capillary loops, *ESKD* end-stage kidney disease, *RAS* renin-angiotensin system

* *P* compare between the IgA groups

^a^ *P* compare with (−) group

^b^ *P* compare with the Mesangial-only group

# No statistical difference in the results of post hoc comparisons using the Bonferroni method to correct significance levels

Statistically significant at *P* < 0.05

The patients with immune deposits in the GCLs-only group were excluded due to small sample size

**Table 9:** Univariate and multivariate analysis of ESKD or ≥ 50% decline in the eGFR

Parameters	Univariate analysis	*P*	Multivariate analysis	*P*
	*HR (95% CI)*		*HR (95% CI)*	
Gender (Female vs Male)	1.476 (0.781, 2.790)	0.231		
Age (years)	1.000 (0.972,1.029)	0.991		
Weight (kg)	1.010 (0.982,1.038)	0.502		
SBP (mmHg)	1.030 (1.015,1.045)	< 0.001	1.026 (1.001, 1.052)	0.041
DBP (mmHg)	1.030 (1.005,1.054)	0.016	0.974 (0.940, 1.009)	0.137
Hemoglobin (g/L)	0.979 (0.964,0.994)	0.006	0.990 (0.969,1.012)	0.368
Serum albumin (g/L)	0.970 (0.935,1.005)	0.089		
eGFR (ml/min/1.73m^2^)	0.954 (0.941, 0.967)	< 0.001	0.959 (0.944, 0.974)	< 0.001
Serum creatinine (μmol/L)	1.021 (1.017,1.025)	< 0.001		
Triglyceride (mmol/L)	1.148 (1.064,1.240)	< 0.001	1.181 (1.025,1.362)	0.022
Cholesterol (mmol/L)	1.029 (0.906,1.168)	0.665		
Serum IgA (g/L)	0.974 (0.716,1.326)	0.869		
Serum IgG (g/L)	0.916 (0.836,1.004)	0.062		
Serum IgM (g/L)	1.389 (0.956,2.018)	0.085		
Serum C3 (g/L)	2.803 (1.146,6.857)	0.024	1.426 (0.373,5.366)	0.609
Proteinuria (g/24 h)	1.171 (1.073,1.279)	< 0.001	1.100 (0.958, 1.263)	0.177
M (1 vs 0)	2.323 (0.318, 16.959)	0.406		
E (1 vs 0)	1.767 (0.424, 7.366)	0.434		
S (1 vs 0)	3.152 (1.226, 8.105)	0.017	1.857 (1.022, 5.127)	0.233
T (0 vs 1 + 2)	12.370 (1.695, 90.262)	0.013	6.754 (0.872, 52.286)	0.067
C (0 vs 1 + 2)	1.198 (0.623,2.302)	0.588		
C1q (−)	1 (reference)		1 (reference)	
Mesangial-only	3.095 (1.195,8.017)	0.020	1.757 (0.595, 5.185)	0.308
Mesangial +GCLs	3.249 (0.984,10.724)	0.053	3.898 (1.022, 14.872)	0.046

Abbreviations: *SBP* systolic blood pressure, *DBP* diastolic blood pressure, *eGFR* estimated glomerular filtration rate, *C1q (−)* without C1q deposits, *GCLs* glomerular capillary loops, *M* mesangial hypercellularity, *E* endocapillary hypercellularity, *S* segmental glomerulosclerosis, *T* tubular atrophy/interstitial fibrosis, *C* Cellular or fibrocellular crescents, *ESKD* end-stage kidney disease

Statistically significant at *P* < 0.05

## Discussion

In this retrospective study, we comprehensively described the clinical and histopathologic findings with respect to the pattern of immunofluorescence deposits in IgAN patients. Compared to patients without C1q deposition, the prognosis in patients with C1q deposition in the mesangial+GCLs group was worsened. However, this prognosis was no different vis-à-vis immunofluorescence deposition in either the mesangial-only group or the mesangial+GCLs in patients with IgAN.

It is conventional medical wisdom that IgA deposits are typically detected in the mesangial area by immunofluorescence, and that IgA deposits extending to the GCLs are observed in 15–50% of IgAN patients [9]. Investigators have reported that IgA deposits localized to the GCLs were closely related to heavier persistent proteinuria and more frequent crescents [10, 11]; these results are partially consistent with our findings. Kobayashi et al. showed that deposits of IgA in the GCLs were observed more commonly in patients with unfavorable outcomes [12]. Similarly, D’Amico et al. reported that IgA deposits extending to the GCLs constituted one of the risk factors for progression [13]. Deposition of immune complexes that consisted of glycan-specific IgG antibodies and galactose-deficient IgA1 in the kidney resulted in kidney injury, perhaps explaining why IgA deposits extended to GCLs are associated with worsened clinicopathologic damage and prognoses. Our results also showed that IgA deposits extending to the GCLs were more likely to be accompanied by IgG deposits. In addition, Katafuchi and colleagues reported that prognoses were better in patients with a higher intensity of IgA deposits compared with those with lower intensity in patients treated with corticosteroids [6]. In a recent study, IgA deposition along the capillary walls was associated with greater histologic severity, including higher mesangial and endocapillary hypercellularity; however, these authors reported that renal survival was comparable regardless of presence or absence of IgA staining in the capillary walls [14]. Our results likewise revealed that the prognosis was not significantly different among groups, but treatment such as with corticosteroids cannot be ignored as a confounding factor. Based on the above reports, we suggest that IgA deposition extending to the GCLs is associated with worsened clinicopathologic findings, although its prognostic significance remains unclear.

The IgG immune complexes bound to aberrantly glycosylated IgA1—and deposited in the glomeruli—result in renal injury [15]; however, the impact of IgG deposition on IgAN is controversial. Christina et al. reported that mesangial staining for IgA and IgG (AG) was associated with hypertension and decreased renal survival [16]; while Wada et al. remarked that the proteinuria was more severe, and the presence of IgA deposition on capillary walls was more frequent in patients with AG deposition than without IgG deposition [17]. In our study, patients with IgG deposition in the mesangial area had lower CHOL levels and milder proteinuria compared to those with IgG in the mesangial area and GCLs, and a lower frequency of T(1 + 2) compared to patients without IgG deposition. The subjects in our study also presented with rather mild baseline features. These results indicated that mesangial IgG deposition might occur more frequently in the early phases of IgA nephropathy, even though the patients with IgG deposition in the mesangial area and GCLs presented with greater clinicopathologic damage. We speculate that IgG deposition extended to the GCLs aggravates the disease process. A study from the UK revealed glomerular IgG deposition was correlated with severe mesangial and endocapillary cellularity [14], and our results confirmed that the pattern of IgG deposits was associated with the M1. Korean investigators stated that moderate and marked glomerular IgG deposition was associated with adverse outcomes; notably, they demonstrated that the pattern of IgG staining (mesangial-only, mesangial + capillary wall, and capillary wall-only) was not connected with either blood pressure or eGFR at biopsy, nor with renal outcomes at follow-up [18]. We also herein showed that the location of IgG deposition exhibited no relationship with blood pressure or GFR at diagnosis. We initially demonstrated that patients with IgG deposition in the mesangial+GCLs group presented with higher levels of CHOL and heavier proteinuria compared with the mesangial-only group. A recent study indicated that the co-deposition of IgG did not affect the renal outcome in IgAN; however, more importantly, these authors suggested that the immunofluorescence deposits extending to the GCLs did influence renal outcome [19]. In our study, the pattern of IgG deposition was not significantly correlated with renal outcomes, which is congruent with the aforementioned Korean study [18]. Over 2/3 (68.8%) of the patients with IgG deposition in our mesangial-only group presented with a higher intensity of IgA deposits (≥3+), while the intensity of IgA deposits was evenly distributed in those individuals with IgG deposition in the mesangial+GCLs group. Compared with patients without IgG deposition, patients with IgG deposition in the mesangial area and GCLs manifested conditions that were more often accompanied by Fib and C1q deposition. However, the difference was not statistically significant compared with patients whose deposits were solely in the mesangial area. Over half (53.1%) of the patients with IgG deposition in the mesangial-only group had a higher intensity of C3 deposition (≥2 +), but approximately half of the patients with IgG deposition in the mesangial+GCLs group exhibited no C3 deposition. These results likely explained why the patients with IgG deposition in the mesangial+GCLs group had prognoses similar to those patients with IgG deposition in the mesangial-only group.

The precise mechanism subserving IgM deposition in IgAN remains unclear, although some investigators postulate that IgM deposition is non-specific in patients with IgAN. As is currently understood, IgM is the first antibody synthesized and secreted in response to infection, and can be converted to IgG by T-cells. The switch of IgM to IgG, then, might be influenced by a dysfunction of T-cells, resulting in IgM deposition [20, 21]. The depletion of B cells was shown to attenuate the deposition of IgM, slowing the rate of progression and relieving the severity of the disease [22]. Our study confirmed that IgM deposition either in the mesangial-only group or in the mesangial+GCLs group was associated with higher serum IgM levels compared to individuals without IgM deposition. Extant kidney damage might be further aggravated due to the deposition of IgM via complement activation [23]. In our study, the intensity of C3 deposition was different depending upon the localization of IgM deposition, and IgM deposition in the mesangial+GCLs group was often accompanied by C1q deposition. Based upon the above observations, we suspected that IgM deposition played an important role in the progression of IgAN. Cihan and colleagues reported that mesangial IgM deposition was associated with greater pathology and an unfavorable renal outcome [24]; however, the significance of IgM deposition in GCLs is arcane. We indicated that an over-release of cytokines would lead to an increase in capillary permeability, resulting in the extension of IgM deposits to the GCLs. The pattern of IgM deposition we observed was then associated with the intensity of IgA deposits and the co-deposition of Fib and C1q, which provides the possibility that the immune deposits also consisted of IgM. However, no difference was displayed between the mesangial+GCLs group and the mesangial-only group regarding IgM in our study. More importantly, the pattern of IgM did not affect the prognosis of IgAN patients.

It is well known that immune-complex deposition caused complement activation [25]. For example, Yasar et al. regarded moderate and marked mesangial C3 deposition as a good predictor of IgAN progression [26], and Korean clinicians observed that mesangial C3 deposition was involved in renal impairment, heavier proteinuria, greater glomerular sclerosis, and interstitial fibrosis [27]. Nasri et al. indicated an association between C3 deposits and serum creatinine, endocapillary proliferation, and segmental glomerulosclerosis [28]. Mesangial C3 deposition combined with the Oxford classification also improved the predictive utility of the IgAN prognosis [29]. Lai and colleagues hypothesized that C3 deposition in the mesangial region and GCLs causes podocyte injury and results in the formation of proteinuria—and proteinuria appeared to be more severe in patients with C3 deposition extending to the GCLs in our study [2]. In a Japanese cohort, researchers showed that the renal outcome was adverse in IgAN patients with extraglomerular C3 deposits [30]. In our study, we failed to find an association between prognosis and the pattern of C3 deposition. Their differing results from our study may be due to the spatial arrangement of immunofluorescence deposits. MUDA et al. showed that patients with severely pathologic damage were more likely to exhibit larger C3 deposits or C3 deposits without an outer coat of IgA [31]. Glomerular C3 deposition was also more frequently present with a higher intensity of IgA deposits. As C3 deposition in the mesangial+GCLs group was more frequently associated with IgG, IgM, Fib, and C1q deposits; we suggest that the spatial arrangement of immunofluorescence deposits be taken into consideration.

Although C1q deposition was found in 2.4–17% of IgAN patients, the significance of C1q deposition remains obscure [6]. The presence of C1q staining associated with autoimmune diseases such as lupus nephritis should therefore receive more attention in IgAN. Thus, we excluded the patients whose condition was combined with other autoimmune diseases. Hiroki et al. reported that C1q deposition was closely related to remission of both proteinuria and hematuria [32]. Ritsuko et al. provided a comprehensive evaluation of C1q deposition in IgAN, revealing that C1q deposition was correlated with the presence of crescent formation, and segmental and global sclerosis [6]. Lee et al. also reported that the presence of mesangial C1q staining was connected to an adverse prognosis. Most importantly, the more marked the IgA deposition, the more frequent the IgG and IgM co-depositions were found in patients with mesangial C1q deposition [33]. In our study, we initially showed clinicopathologic features of those patients with C1q deposition in the mesangial region and GCLs. We observed that the frequency of IgA deposition ≥3+ was more common in patients with C1q deposition in the mesangial region and GCLs, with the frequencies of IgG, IgM, and Fib co-deposition at 57.1, 78.6, and 35.7%, respectively. Liu et al. also observed that C1q deposition was more frequent in patients with severe renal impairment [34]. In our study, Kaplan–Meier and Cox regression analyses illustrated that C1q deposition in the mesangial area and GCLs predicted a poor renal prognosis. However, we could not draw a clear conclusion regarding the significance of C1q deposition in IgAN patients due to the small number of patients with this characteristic.

Fib deposits are rarely seen in the glomeruli under normal physiologic conditions. Many scholars posit that extravascular tissue factors activate coagulation factors under inflammatory conditions, leading to Fib deposition [35]. Although Haas has suggested that Fib deposits in the mesangial region possess a granular appearance, staining in the GCLs was nonspecific [36]. In our study, we showed that the pattern of Fib deposits was related to the intensity of IgA deposits and the presence of IgG, IgM, and C1q deposits. These results highlighted the importance of intra-mesangial coagulation in IgAN, and it is therefore of great importance to investigate the mechanism(s) underlying Fib deposition in IgAN.

There were some limitations to our study. First, the number of samples was relatively small and the follow-up time was brief. Second, our conclusions cannot be generalized to other racial or ethnic groups due to this being a single-center study. Additionally, we did not perform IgG subtyping at biopsy. Third, the patients with deposition only in the GCLs were excluded due to their limited number. Finally, some factors that may affect a prognosis were not taken into consideration, including the spatial arrangement of immunofluorescence deposits and treatments that might change during follow-up.

## Conclusions

In this study, we explored the different roles for patterns of immunofluorescence deposits in IgAN by conducting a retrospective analysis. The location of glomerular IgA, IgG, IgM, C3, and Fib deposits did not affect the prognosis of IgAN patients—except for C1q deposits. This study strengthened the significance of immunofluorescence deposits, and provides a more comprehensive prognostic evaluation system for IgAN.

## Data Availability

Datasets used and/or analyzed during the current study are available from the corresponding author on reasonable request.

## Abbreviations

SBP: Systolic blood pressure
DBP: Diastolic blood pressure
MAP: Mean arterial pressure
eGFR: Estimated glomerular filtration rate
CHOL: Cholesterol
ESKD: End-stage kidney disease
Fib: Fibrinogen
GCLs: Glomerular capillary loops
M: Mesangial hypercellularity
S: Segmental sclerosis
T: Interstitial fibrosis/tubular atrophy
C: Crescent
M0: Mesangial hypercellularity score of ≤0.5
M1: Mesangial hypercellularity score >0.5
E0: Absence of endocapillary hypercellularity
E1: Presence of endocapillary hypercellularity
S0: Absence of segmental glomerulosclerosis
S1: Presence of segmental glomerulosclerosis
T0: Tubular atrophy/interstitial fibrosis ≤25% of cortical area
T1: Tubular atrophy/interstitial fibrosis 26–50% of cortical area
T2: Tubular atrophy/interstitial fibrosis>50% of cortical area
C0: No crescent in glomeruli
C1: Crescent in < 25% of glomeruli
C2: Crescent in ≥ 25% of glomeruli

## References

[1] Moriyama T, Tanaka K, Iwasaki C, Oshima Y, Ochi A, Kataoka H, Itabashi M, Takei T, Uchida K, Nitta K (2014). Prognosis in IgA nephropathy: 30-year analysis of 1,012 patients at a single center in Japan. PLoS One.

[2] Lai KN, Tang SC, Schena FP, Novak J, Tomino Y, Fogo AB, Glassock RJ (2016). IgA nephropathy. Nat Rev Dis Primers.

[3] Berthoux F, Mohey H, Laurent B, Mariat C, Afiani A, Thibaudin L (2011). Predicting the risk for dialysis or death in IgA nephropathy. J Am Soc Nephrol.

[4] Le W, Liang S, Hu Y, Deng K, Bao H, Zeng C, Liu Z (2012). Long-term renal survival and related risk factors in patients with IgA nephropathy: results from a cohort of 1155 cases in a Chinese adult population. Nephrol Dial Transplant.

[5] Markowitz G (2017). Glomerular disease: updated Oxford classification of IgA nephropathy: a new MEST-C score. Nat Rev Nephrol.

[6] Katafuchi R, Nagae H, Masutani K, Tsuruya K, Mitsuiki K (2019). Comprehensive evaluation of the significance of immunofluorescent findings on clinicopathological features in IgA nephropathy. Clin Exp Nephrol.

[7] Liu D, You J, Liu Y, Tang X, Tan X, Xia M, Wu L, Chen G, He L, Zhu X, Liu H (2019). Serum immunoglobulin G provides early risk prediction in immunoglobulin a nephropathy. Int Immunopharmacol.

[8] Levey AS, Stevens LA, Schmid CH, Zhang YL, Castro AF, Feldman HI, Kusek JW, Eggers P, Van Lente F, Greene T, Coresh J, Ckd EPI (2009). A new equation to estimate glomerular filtration rate. Ann Intern Med.

[9] Parai SK, Ghose T (1985). IgA nephropathy in adults: immunohistologic findings and clinical course. Can Med Assoc J.

[10] Vangelista A, Frasca GM, Mondini S, Bonomini V (1984). Idiopathic IgA mesangial nephropathy: immunohistological features. Contrib Nephrol.

[11] Yoshimura M, Kida H, Abe T, Takeda S, Katagiri M, Hattori N (1987). Significance of IgA deposits on the glomerular capillary walls in IgA nephropathy. Am J Kidney Dis.

[12] Kobayashi Y, Tateno S, Hiki Y, Shigematsu H (1983). IgA nephropathy: prognostic significance of proteinuria and histological alterations. Nephron.

[13] D'Amico G, Minetti L, Ponticelli C, Fellin G, Ferrario F, Barbiano di Belgioioso G, Imbasciati E, Ragni A, Bertoli S, Fogazzi G (1986). Prognostic indicators in idiopathic IgA mesangial nephropathy. Q J Med.

[14] Bellur SS, Troyanov S, Cook HT, Roberts IS, A.N.N (2011). Working Group of International Ig, S. Renal Pathology, Immunostaining findings in IgA nephropathy: correlation with histology and clinical outcome in the Oxford classification patient cohort. Nephrol Dial Transplant.

[15] Floege J (2011). The pathogenesis of IgA nephropathy: what is new and how does it change therapeutic approaches?. Am J Kidney Dis.

[16] Nieuwhof C, Kruytzer M, Frederiks P, van Breda Vriesman PJ (1998). Chronicity index and mesangial IgG deposition are risk factors for hypertension and renal failure in early IgA nephropathy. Am J Kidney Dis.

[17] Wada Y, Ogata H, Takeshige Y, Takeshima A, Yoshida N, Yamamoto M, Ito H, Kinugasa E (2013). Clinical significance of IgG deposition in the glomerular mesangial area in patients with IgA nephropathy. Clin Exp Nephrol.

[18] Shin DH, Lim BJ, Han IM, Han SG, Kwon YE, Park KS, Lee MJ, Oh HJ, Park JT, Han SH, Kang SW, Yoo TH (2016). Glomerular IgG deposition predicts renal outcome in patients with IgA nephropathy. Mod Pathol.

[19] Alvarado AS, Andeen NK, Brodsky S, Hinton A, Nadasdy T, Alpers CE, Blosser C, Najafian B, Rovin BH (2018). Location of glomerular immune deposits, not codeposition of immunoglobulin G, influences definitive renal outcomes in immunoglobulin a nephropathy. Nephrol Dial Transplant.

[20] Lin CY, Chen CH, Lee PP (1989). In vitro B-lymphocyte switch disturbance from IgM into IgG in IgM mesangial nephropathy. Pediatr Nephrol.

[21] Lin CY, Chu CM (1986). Studies of circulating immune complexes and lymphocyte subpopulations in childhood IgM mesangial nephropathy. Nephron.

[22] Strassheim D, Renner B, Panzer S, Fuquay R, Kulik L, Ljubanovic D, Holers VM, Thurman JM (2013). IgM contributes to glomerular injury in FSGS. J Am Soc Nephrol.

[23] Platt JL, Cascalho M (2015). IgM in the kidney: a multiple personality disorder. Kidney Int.

[24] Heybeli C, Oktan MA, Yildiz S, Arda HU, Unlu M, Cavdar C, Sifil A, Celik A, Sarioglu S, Camsari T (2019). Clinical significance of mesangial IgM deposition in patients with IgA nephropathy. Clin Exp Nephrol.

[25] Al Hussain T, Hussein MH, Al Mana H, Akhtar M (2017). Pathophysiology of IgA nephropathy. Adv Anat Pathol.

[26] Caliskan Y, Ozluk Y, Celik D, Oztop N, Aksoy A, Ucar AS, Yazici H, Kilicaslan I, Sever MS (2016). The clinical significance of uric acid and complement activation in the progression of IgA nephropathy. Kidney Blood Press Res.

[27] Kim SJ, Koo HM, Lim BJ, Oh HJ, Yoo DE, Shin DH, Lee MJ, Doh FM, Park JT, Yoo TH, Kang SW, Choi KH, Jeong HJ, Han SH (2012). Decreased circulating C3 levels and mesangial C3 deposition predict renal outcome in patients with IgA nephropathy. PLoS One.

[28] Nasri H, Sajjadieh S, Mardani S, Momeni A, Merikhi A, Madihi Y, Ghiessari A, Emami Naieni A (2013). Correlation of immunostaining findings with demographic data and variables of Oxford classification in IgA nephropathy. J Nephropathol.

[29] Park S, Kim HW, Park JT, Chang TI, Kang EW, Ryu DR, Yoo TH, Chin HJ, Jeong HJ, Kang SW, Lim BJ, Han SH (2020). Relationship between complement deposition and the Oxford classification score and their combined effects on renal outcome in immunoglobulin A nephropathy. Nephrol Dial Transplant..

[30] Ohsawa I, Kusaba G, Ishii M, Sato N, Inoshita H, Onda K, Hashimoto A, Nagamachi S, Suzuki H, Shimamoto M, Ohi H, Horikoshi S, Tomino Y (2013). Extraglomerular C3 deposition and metabolic impacts in patients with IgA nephropathy. Nephrol Dial Transplant.

[31] Muda AO, Feriozzi S, Rahimi S, Faraggiana T (1995). Spatial arrangement of IgA and C3 as a prognostic indicator of IgA nephropathy. J Pathol.

[32] Nishiwaki H, Hasegawa T, Nagayama Y, Kaneshima N, Takayasu M, Hirose M, Komukai D, Inoue Y, Koiwa F, Yoshimura A (2015). Absence of mesangial C1q deposition is associated with resolution of proteinuria and hematuria after tonsillectomy plus steroid pulse therapy for immunoglobulin a nephropathy. Nephron.

[33] Lee HJ, Choi SY, Jeong KH, Sung JY, Moon SK, Moon JY, Lee SH, Lee TW, Ihm CG (2013). Association of C1q deposition with renal outcomes in IgA nephropathy. Clin Nephrol.

[34] Liu Y, Hu Q, Shen P, Tang L, Yuan G, Zhou Y, Chai H (2016). Clinical and pathological analysis of IgA nephropathy with chronic renal failure. Ren Fail.

[35] He Z, Zhang Y, Cao M, Ma R, Meng H, Yao Z, Zhao L, Liu Y, Wu X, Deng R, Dong Z, Bi Y, Kou J, Novakovic V, Shi J, Hao L (2016). Increased phosphatidylserine-exposing microparticles and their originating cells are associated with the coagulation process in patients with IgA nephropathy. Nephrol Dial Transplant.

[36] Haas M (2005). Histology and immunohistology of IgA nephropathy. J Nephrol.

